# Regulatory element map of sheep reproductive tissues: functional annotation of tissue-specific strong active enhancers

**DOI:** 10.3389/fvets.2025.1564148

**Published:** 2025-04-16

**Authors:** Zhu Meng, Mingxing Chu, Hao Yang, Shiwen Zhang, Qiangjun Wang, Jiahong Chen, Chunhuan Ren, Zhangyuan Pan, Zijun Zhang

**Affiliations:** ^1^Anhui Provincial Key Laboratory of Conservation and Germplasm Innovation of Local Livestock, College of Animal Science and Technology, Anhui Agricultural University, Hefei, China; ^2^State Key Laboratory of Animal Biotech Breeding, Institute of Animal Science, Chinese Academy of Agricultural Sciences, Beijing, China; ^3^National Nanfan Research Institute (Sanya), Chinese Academy of Agricultural Sciences, Sanya, China

**Keywords:** sheep, regulatory element, tissue specific, enhancer, reproductive tissues

## Abstract

**Introduction:**

Comprehensive functional annotation of the genome is crucial for elucidating the molecular mechanisms underlying complex traits and diseases. Although functional annotation has been partially completed in sheep, a systematic annotation focused on reproductive tissues remains absent.

**Methods:**

In this study, we integrated 60 transcriptomic and epigenomic datasets from five reproductive tissues. Using a multi-omics approach, we predicted 15 distinct chromatin states and conducted thorough functional annotation.

**Results:**

We established the first regulatory element atlas for sheep reproductive tissues and examined the roles of these elements in reproductive traits and disease. In total, we annotated 1,680,172 regulatory elements, including 83,980 tissue-specific strong active enhancers (EnhAs).

**Discussion:**

Enhancers were identified as critical drivers of tissue-specific functions, operating through sequence-specific transcription factor binding and direct regulation of target genes. Key transcription factors associated with reproductive function included *INHBA* (ovary), *KITLG* (oviduct), *Snai2* (cervix), *WNT7A* (uterine horn), *FOLR1* (uterine body), and *SALL1* (shared uterine regions). Additionally, our findings support the potential of sheep as a promising model for investigating embryonic development and miscarriage. This work lays a theoretical foundation for future research into the molecular mechanisms of complex traits and diseases in sheep.

## 1 Introduction

Sheep are one of the most important domesticated animals and play a crucial role in ensuring food and livestock product security. Reproductive traits, as critical economic indicators, directly affect production costs and economic returns of the sheep farming industry (1). Studies have demonstrated that the uterus (2–4), ovaries (5), and oviducts (6) are the primary organs influencing sheep reproductive performance.

Functional annotation of regulatory elements in the major organs of domesticated animals is crucial for understanding the molecular mechanisms of economically important complex traits, including growth, reproduction, and disease resistance (7, 8). Gene expression in specific tissues and cells, along with their physiological roles, is regulated by cis-regulatory elements such as enhancers and promoters. These elements control the expression patterns of target genes by recruiting sequence-specific transcription factors (TFs) in a tissue-specific manner (9, 10). Therefore, systematically analyzing the functions of tissue-specific regulatory elements in the sheep uterus, ovaries, and oviducts is crucial for understanding the physiological roles of reproductive tissues. Genome-wide association studies (GWAS) have identified a large number of gene variants associated with diseases and complex traits (11). However, over 90% of single-nucleotide polymorphisms (SNPs) identified in GWAS loci are located in noncoding regions, indicating that the activity of noncoding regions is a primary driver of phenotypic variation (12, 13). Noncoding variants are mainly concentrated in regulatory elements. Thus, comprehensive annotation of functional regulatory elements, especially in noncoding regions, will contribute to resolving fundamental biological questions (14), uncovering the genetic architecture of disease risk and phenotypic variation (15), and improving the accuracy of polygenic trait predictions (1, 16).

The Encyclopedia of DNA Elements (ENCODE) project, the Roadmap Epigenomics Project, and the Functional Annotation of Animal Genomes (FAANG) consortium have clearly demonstrated the importance of regulatory element annotation in humans, model organisms, and domesticated animals (7, 8, 17). After years of effort, regulatory element maps have been identified and annotated for humans (17, 18), mice (19), zebrafish (20), dogs (21), pigs (22), chickens (23), cattle (24), and horses (25), with continual refinement of these datasets. However, the annotation of regulatory elements in sheep has been completed for only a few tissues (26, 27), with annotations for tissues related to reproductive traits still missing. In this study, 40 CUT&Tag, 10 ATAC-Seq, and 10 RNA-Seq datasets generated by the authors’ laboratory were utilized. The datasets were derived from reproductive tissues of sheep, including uterus (cervix, cornua uteri, and corpus uteri), ovaries, and oviduct tissues. Through a multi-omics integrative analysis, we identified 15 distinct chromatin states and investigated the functions of tissue-specific regulatory elements. Furthermore, association analyses between these regulatory elements and phenotypic data from humans and mice were performed. The ultimate objective was to construct a comprehensive map of regulatory elements in sheep reproductive tissues, demonstrating their roles in influencing reproductive traits and diseases in sheep.

## 2 Materials and methods

### 2.1 Animals and tissues

Tissue collection was carried out following protocols approved by the Animal Welfare and Ethics Committee of the Institute of Animal Science, Chinese Academy of Agricultural Sciences, Beijing. Two female Small-Tailed Han sheep, each from different families, were selected. The ewes were 3 years old, sexually mature, and had given birth twice. The ewes underwent estrus synchronization and were euthanized 24 h after estrus detection. All experimental animals were euthanized via intravenous injection of pentobarbital sodium at a dose of 100 mg/kg. Five reproductive tissues were collected: the cervix, cornua uteri, corpus uteri, ovaries, and oviducts. All tissue samples were isolated in strict accordance with the standards of the FAANG project. The isolated tissues were immediately flash-frozen in liquid nitrogen and then stored in a − 80°C freezer for long-term preservation for future use.

### 2.2 Library construction and sequencing

The frozen tissues collected in the previous step were selected for RNA sequencing (RNA-seq), Assay for Transposase-Accessible Chromatin using sequencing (ATAC-seq), and Cleavage Under Targets and Tagmentation (CUT&Tag) experiments. The CUT&Tag experiment includes four histone modification markers: H3K4me3, H3K27ac, H3K4me1, and H3K27me3. H3K4me3 is highly enriched at active promoters near transcription start sites (TSS) and shows a positive correlation with transcription (28). H3K27ac is found in both the proximal and distal regions of transcription start sites (TSS) and is considered a marker of active enhancers (29). H3K4me1 is enriched in both active and primed enhancers and plays a critical role in regulating the expression of cell identity genes, making it essential for determining cell identity (30). H3K27me3 represses gene expression and shares a location with H3K27ac, with the two marks interacting in an antagonistic manner (31). For RNA-seq, total RNA was extracted using TRIzol reagent, and its integrity was assessed to ensure a minimum RNA integrity number (RIN) value of 6. rRNA was then removed to purify the samples, and strand-specific RNA-seq libraries were constructed using the dUTP method following Illumina’s protocol. The libraries were sequenced on the Illumina HiSeq X Ten PE150 platform. For ATAC-seq, the isolation of frozen tissue nuclei was undertaken employing the 52201-10 protocol. Approximately 5 mg of tissue sample was added to 0.5 mL of lysis buffer, and the sample was ground with a pestle until no visible clumps remained. The mixture was incubated on ice for 2–10 min, filtered to remove impurities, and resuspended in suspension buffer, yielding 50,000 nuclei. The Hyperactive ATAC-Seq Library Prep Kit for Illumina was then used to prepare the ATAC-seq library. Nuclei were incubated in a transposase reaction mix at 37°C for 30 min. DNA was extracted using ATAC DNA Extract Beads, followed by amplification and two-step size selection with ATAC DNA Clean Beads. The resulting DNA fragments were quantified and sequenced on the Illumina HiSeq X Ten PE150 platform. For CUT&Tag, nuclei were extracted using the same method as in the ATAC-seq experiment. The construction of the library was then performed using the Hyperactive Universal CUT&Tag Assay Kit for Illumina Pro. Extracted nuclei were incubated overnight at 4°C with Diagenode primary antibodies (0.4 μL H3K4me3, 0.66 μL H3K4me1, 0.4 μL H3K27ac, and 0.9 μL H3K27me3). The samples were then incubated at room temperature with a rabbit secondary antibody for 1 h, followed by incubation with pA/G-Tnp Pro for another hour. Magnesium ions were added to activate the transposase for DNA fragmentation. The fragmented DNA was purified using VAHTS DNA Clean Beads, and the purified fragments were quantified and sequenced on the Illumina HiSeq X Ten PE150 platform.

### 2.3 Raw sequence data processing

The ATAC-seq and RNA-seq data were processed using the FAANG functional annotation pipeline developed by the University of California, Davis.^1^ The CUT&Tag data analysis process followed the methodology established by the Steven team.^2^ All analyses were performed using the Ramb_v2.0 sheep reference genome. For data quality control and trimming, Trim Galore (v.0.6.5) was used. RNA-seq reads were aligned to the reference genome using STAR (v.2.7.11a), while ATAC-seq reads were aligned using BWA (v.2.2.1). Samtools (v.1.18) was applied to filter out reads with a mapping quality (MAPQ) score below 30. For RNA-seq, HTSeq-Count (v.2.0.4) was utilized to extract gene read counts, and gene expression levels were normalized with EdgeR (v.4.0.2) and StringTie2 (v.2.2.1). For CUT&Tag data, duplicate reads were removed using Picard (v.3.1.1), and peak calling was performed using MACS2 (v.2.2.9.1). Hi-C data were analyzed using the Juicer software, including the identification of topologically associated domains (TADs).^3^

### 2.4 Sample clustering

Clustering analysis was conducted on all samples across different tissues and sequencing methods using deepTools (v.3.5.0). The tag signals (bigWig) for each sample were generated using bamCompare and subsequently normalized using Z-score through the scipy.stats.zscore function in Scipy (v.1.8.0). The Z-score-normalized signals for all samples were summarized using multiBigwigSummary. Correlation analysis between samples was performed using the plotCorrelation function, and principal component analysis (PCA) was conducted on the samples using the plotPCA function.

### 2.5 Annotation of chromatin states

First, we downloaded the sheep reference genome (fna) and genome annotation file (gtf) from the NCBI website.^4^ Using the STAR software (v2.7.11b), we input the fna and gtf files to generate the CHROMSIZES file for the sheep genome. Then, using gtfToGenePred (v469), we converted the gtf file into a genePred file. Next, with ChromHMM (v1.26) and its ConvertGeneTable function (using default parameters), we input the genePred file to produce sheep genome annotation files in the COORDS and ANCHORFILES directories. After setting up the required files, we used the binarizeBam function (with default parameters) from ChromHMM to process the CUT&Tag and ATAC-seq bam files, generating binary matrices. Finally, we trained a model using the LearnModel function (with default parameters), which resulted in the generation of chromatin states. Fifteen distinct chromatin states were identified based on significant epigenetic marks and selected for annotation. The chromatin states were determined by epigenomic modifications and their enrichment around transcription start sites (TSSs) (17, 32), and the following names are employed: Strongly active promoters/transcripts (TssA), Flanking active TSS without ATAC (TssAHet), Transcribed at gene (TxFlnk), Weakly transcribed at gene (TxFlnkWk), Transcribed region without ATAC (TxFlnkHet), Strong active enhancer (EnhA). Medium enhancer with ATAC (EnhAMe), Weak active enhancer (EnhAWk), Active enhancer without ATAC (EnhAHet), Poised enhancer (EnhPois), ATAC island (ATAC_Is), Bivalent/poised TSS (TssBiv), Repressed polycomb (Repr), Weak repressed polycomb (ReprWk), and Quiescent (Qui). For each chromatin state, the number, size, and gene coverage were calculated to provide a comprehensive annotation.

### 2.6 Identification and differential analysis of tissue-specific regulatory elements

To investigate the EnhA in each tissue type, the number of overlapping regulatory regions across tissue (RRAT) was calculated across tissues. An RRAT region was assigned a value of 1 for a given tissue if at least one EnhA overlapped; otherwise, it was assigned a value of 0. This analysis resulted in the generation of six tissue-specific regulatory (TSR) enhancer modules: a common uterine module (shared across the three uterine tissues) and five distinct tissue-specific modules. The topologically associating domains (TADs) derived from Li Menghua’s Hi-C dataset were utilized to establish a connection between EnhA and potential target genes, thus facilitating the identification of target genes associated with the TSR (33). Gene Ontology (GO) enrichment analysis of these target genes was conducted using clusterProfiler (version 4.4.1). To investigate the impact of sheep tissue-specific EnhAs on human and mouse phenotypes, we downloaded the GCF_016772045.2ToHg38.over.chain.gz file from https://hgdownload.soe.ucsc.edu/hubs/GCF/016/772/045/GCF_016772045.2/liftOver/ and used the Liftover tool to convert sheep tissue-specific EnhAs into human genome segments. GREAT (Genomic Regions Enrichment of Annotations Tool) is a powerful online tool for the functional annotation of genomic regions, encompassing extensive phenotype data for humans and mice. Based on the converted human genome segments, we performed enrichment analysis for human and mouse phenotypes using the GREAT tool (parameters: 2 kb upstream proximally, 1 kb downstream, and an additional 3 kb distally). HOMER (v.4.11) was employed to identify motifs significantly enriched (FDR < 0.05) in tissue-specific EnhAs. The top five motifs with tissue-specific functions were selected as candidate motifs for each tissue.

## 3 Results

### 3.1 Summary of epigenomic data from sheep reproductive tissues

**Figure 1:**
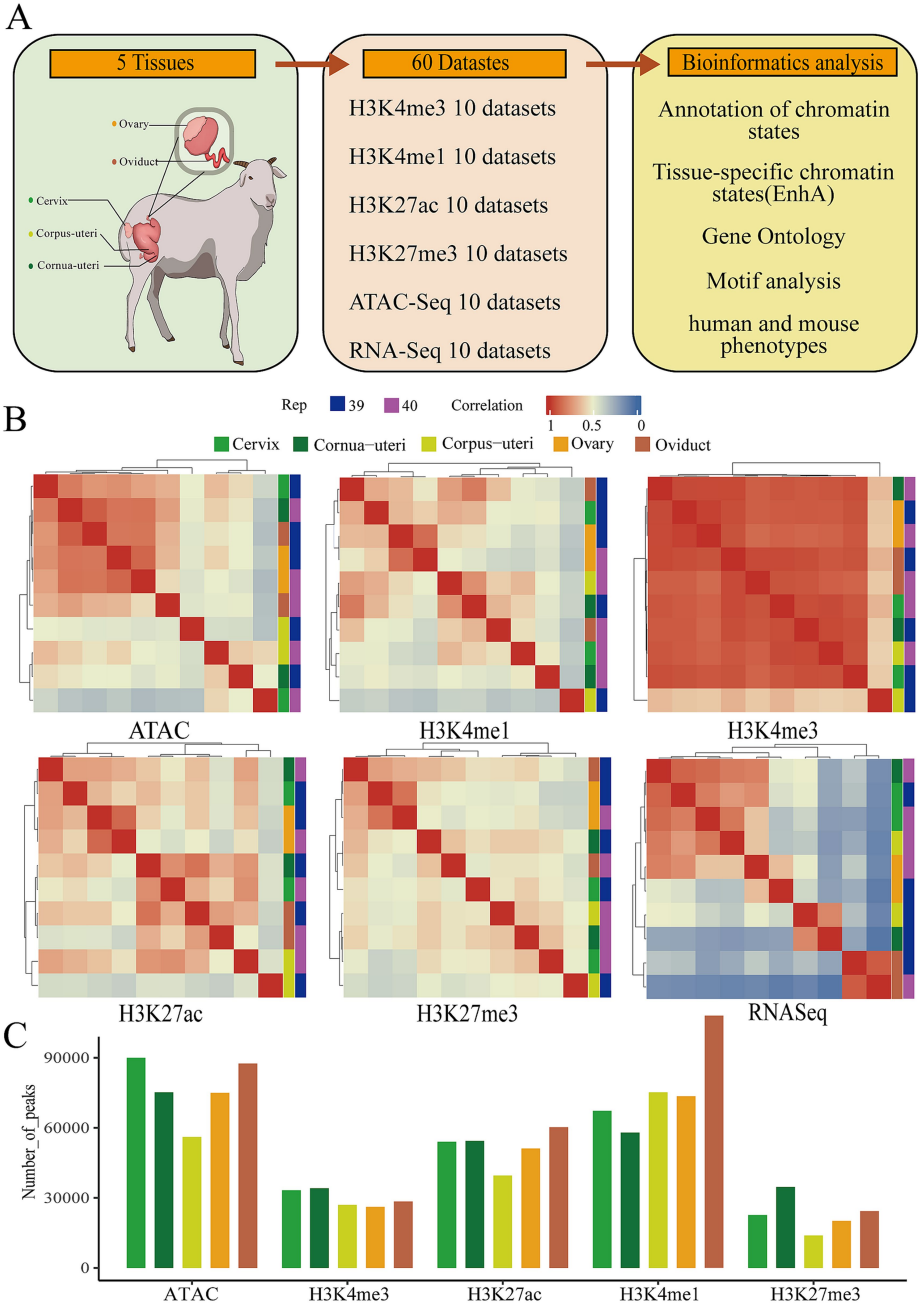
Summary of the data used to construct the epigenomic map of sheep reproductive tissues. **(A)** Schematic diagram of the tissues and datasets analyzed in this study. The left figure shows a schematic of the reproductive tissue types analyzed, the middle displays the number of epigenomic (ATAC-Seq, H3K4me3, H3K4me1, H3K27me3, and H3K27AC) and transcriptomic (RNA-Seq) samples, and the right figure depicts the main bioinformatics and statistical analyses used in the study. **(B)** The relationship between tissues based on the Pearson correlation of their normalized signals within a 1 kb window across the entire genome. **(C)** The average number of peaks for five epigenetic marks across different tissues.

We integrated 60 whole-genome sequencing datasets from five sheep reproductive tissues, which was conducted, encompassing four histone modifications (H3K27ac, H3K27me3, H3K4me3, and H3K4me1) measured using CUT&Tag, along with ATAC-seq and RNA-seq data. Comprehensive bioinformatics analyses were performed to identify and characterize tissue-specific enhancers, chromatin states, and their association with human and mouse phenotypes (Figure 1A). A total of 1.4 billion clean reads were generated, with an average mapping rate of 97.27% (Supplementary Table 1). The analysis yielded an average of 76,753 peaks for ATAC, 51,889 peaks for H3K27ac, 23,111 peaks for H3K27me3, 76,390 peaks for H3K4me3, and 29,782 peaks for H3K4me1 across the five tissues. These peaks covered 1.29, 1.41, 0.48, 2.59, and 1.17% of the entire genome (Figure 1C; Supplementary Table 1; Supplementary Figure 1). In order to evaluate the relationships between different tissues, a hierarchical clustering based on the signal intensity of epigenetic markers and gene expression profiles was performed, followed by principal component analysis (PCA). The results showed that the oviduct was distinct from the ovary and uterus, exhibiting a negative correlation. In contrast, the cervix, cornua uteri, and corpus uteri demonstrated a positive correlation (Figure 1B; Supplementary Figure 2).

### 3.2 Identification and characterization of chromatin states in sheep reproductive tissues

**Figure 2:**
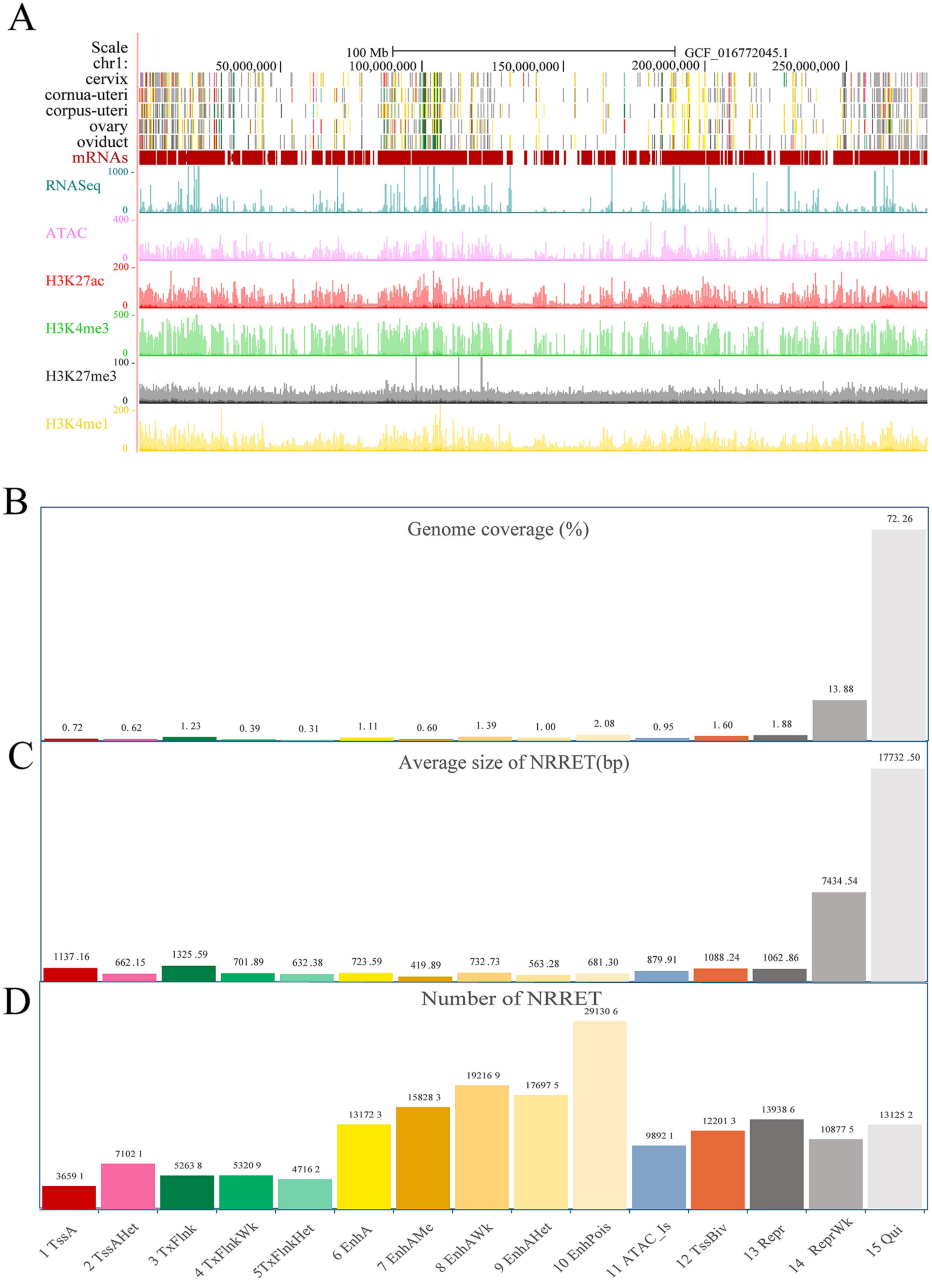
The genome-wide chromatin state landscape across five tissues. **(A)** Visualization on the UCSC browser (chr1), including chromatin states, CUT&Tag, ATAC-seq, and RNA-seq data. **(B)** Genome coverage of each chromatin state (proportion, not percentage). **(C)** The total number of non-redundant regulatory elements (NRRET) and their average size within each chromatin state. **(D)** The total number of NRRET in five sheep reproductive tissues, across each chromatin state.

Utilizing ChromHMM, we integrated the five epigenetic markers across sheep reproductive tissues and identified 15 distinct chromatin states. Chromatin states are classified into six categories based on their function: 1. Promoters, such as TssA, TssAHet, and TssBiv, which make up 2.93% of the entire genome; 2. TSS proximal transcription regions, including TxFlnk, TxFlnkWk, and TxFlnkHet, which make up 1.93% of the entire genome; 3. Enhancers, such as EnhA, EnhAMe, EnhAWk, EnhAHet, and EnhPois, which comprise 6.18% of the genome; 4. ATAC islands, including ATAC_Is, which constitute 0.95% of the genome; 5. Repressive regions, such as ReprWk, which account for 15.75% of the genome; 6. Quiescent regions, including Qui, which occupy 72.26% of the genome (Figure 2B). Across five reproductive tissues, 1,680,172 regulatory elements were identified, excluding Quiescent regions. These elements include 229,625 promoters, with an average size of 962 bp; 153,009 TSS proximal transcription regions, with an average size of 886 bp; 950,456 enhancers, with an average size of 624 bp; 98,921 ATAC islands, with an average size of 879 bp; and 248,161 repressive regions, with an average size of 4,248 bp (Figures 2C,D). To facilitate visualization and exploration, we uploaded datasets for chromatin states, CUT&Tag, ATAC-seq, and RNA-seq to the UCSC Genome Browser, focusing on chromosome 1 (Figure 2A). It was observed that regions exhibiting higher gene density were characterized by active chromatin states, elevated gene expression, and increased chromatin accessibility.

### 3.3 Ovarian tissue-specific EnhA and their functional annotation

**Figure 3:**
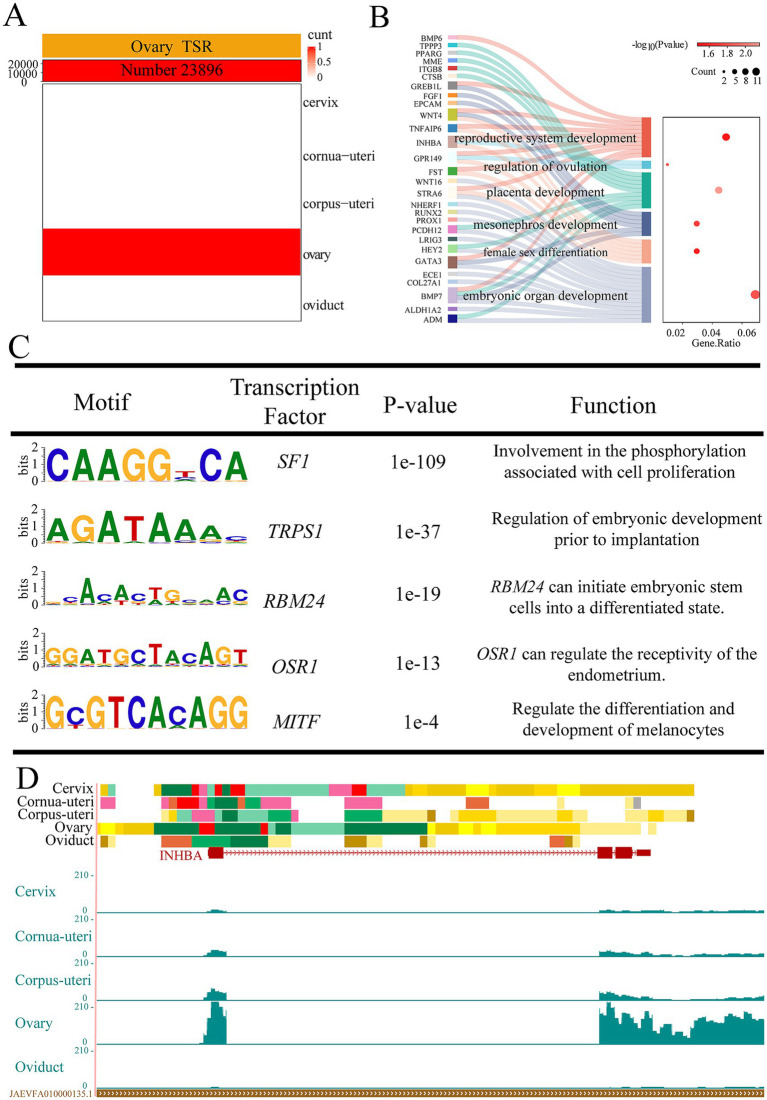
Ovarian tissue-specific strong enhancers (EnhA) and their functional annotation. **(A)** The number and spatial distribution of strong active enhancer (EnhA) in ovarian tissue, along with their enrichment patterns. **(B)** GO functional enrichment analysis based on the target genes of ovarian EnhAs. **(C)** Enrichment analysis of transcription factor motifs in ovarian tissue. **(D)** The chromatin state landscape and mRNA expression of the *INHBA* (chr4:80,959,294-80,976,837, Ramb_v2.0) locus across five tissues. The vertical scale of the UCSC track represents the normalized RNA-seq signal, ranging from 0 to 210.

Our previous research has shown that enhancers demonstrate the greatest variability across different tissues among the 15 chromatin states (23). Building on this, we focused on tissue-specific EnhA and identified 23,896 EnhAs in the ovary (Figure 2A). In order to further explore the biological functions of EnhAs, GO analysis was conducted on their predicted target genes. The result indicates that ovarian-specific EnhAs primarily involved in regulating ovulation, reproductive system development, and placental development. Motifs enrichment analysis of the ovarian tissue-specific EnhAs identified several motifs such as *SF1*, *TRPS1*, *RBM24*, *OSR1*, and *MITF*, all of which are associated with the biological functions of the ovary. Of particular note is the function of *TRPS1*, which has been identified as a primary regulator of embryonic development prior to implantation. Furthermore, *OSR1* has been demonstrated to regulate the receptivity of the endometrium (Figures 3B–C). To exemplify how regulatory elements regulate tissue-specific gene expression, the inhibin subunit beta A (*INHBA*) gene locus was used as a model (Figure 3D). *INHBA* is highly expressed in the ovary, with nearby regulatory elements displaying active chromatin states.

### 3.4 Oviducal tissue-specific EnhA and their functional annotation

**Figure 4:**
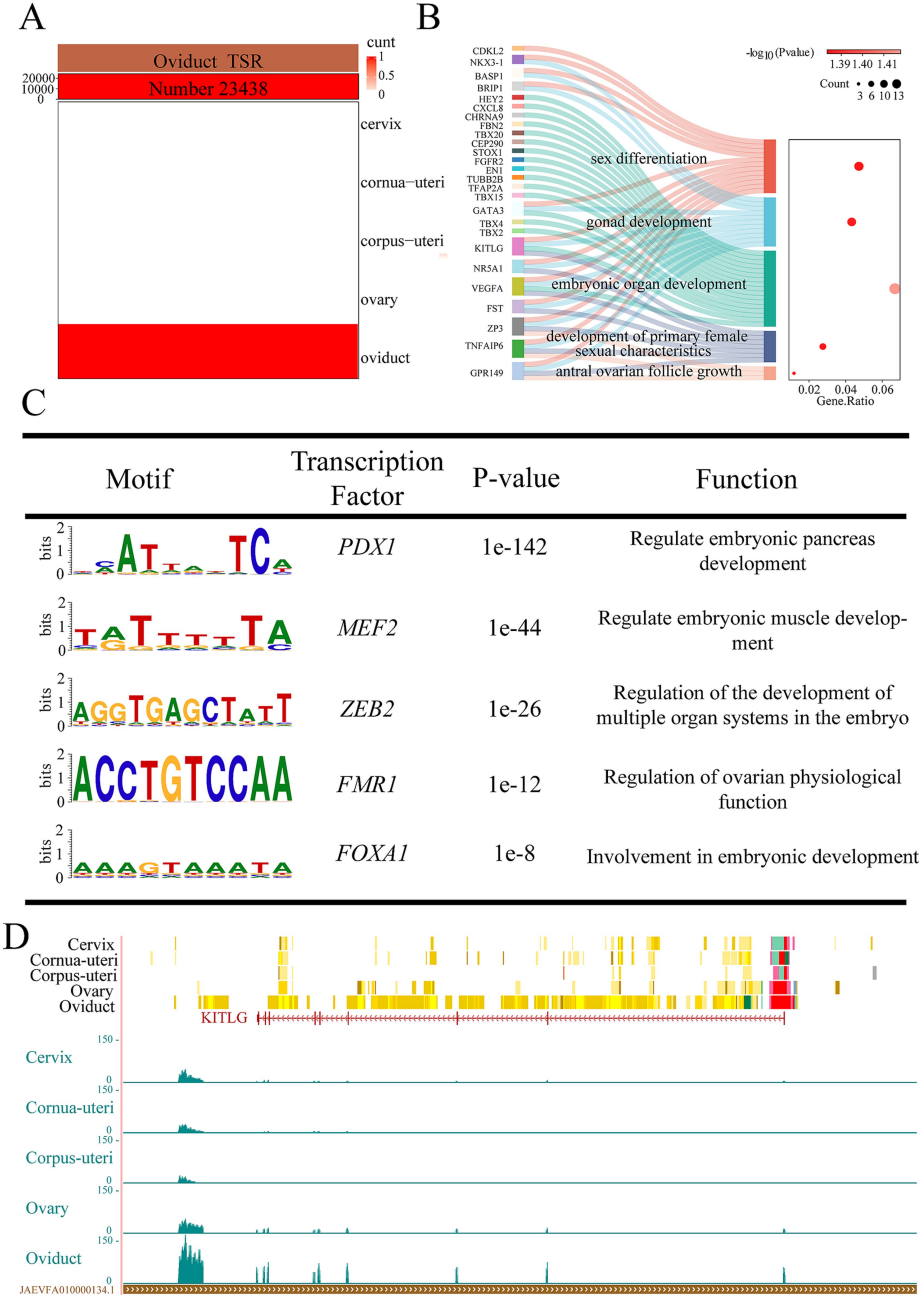
Oviducal tissue-specific strong enhancers (EnhA) and their functional annotation. **(A)** The number and spatial distribution of EnhA in oviducal tissue, along with their enrichment patterns. **(B)** GO functional enrichment analysis based on the target genes of oviducal EnhAs. **(C)** Enrichment analysis of transcription factor motifs in oviducal tissue. **(D)** The chromatin state landscape and mRNA expression of the *KITLG* (chr3:124,751,722-124,891,391, Ramb_v2.0) locus across five tissues. The vertical scale of the UCSC track represents the normalized RNA-seq signal, ranging from 0 to 150.

We identified 23,438 tissue-specific EnhAs in oviducal tissue (Figure 4A). GO analysis of predicted target genes of oviduct-specific EnhAs shows their role in regulating functions of the oviduct, including antral ovarian follicle growth, gonad development, and the development of primary female sexual characteristics (Figure 4B). Motif enrichment analysis identified several transcription factor binding motifs associated with oviducal EnhAs, including *PDX1*, *MEF2*, *ZEB2*, *FMR1*, and *FOXA1*. Among these, *FOXA1* is particularly noteworthy for its potential role in regulating ovarian physiological functions through interactions with the oviduct (Figure 4C). In order to illustrate the role of the oviduct in reproduction, KIT ligand (*KITLG*) was used as an example. As a target gene of EnhAs in the oviduct, *KITLG* exhibits a high expression level in this tissue (Figure 4D).

### 3.5 Uterine common tissue-specific EnhA and functional annotation

**Figure 5:**
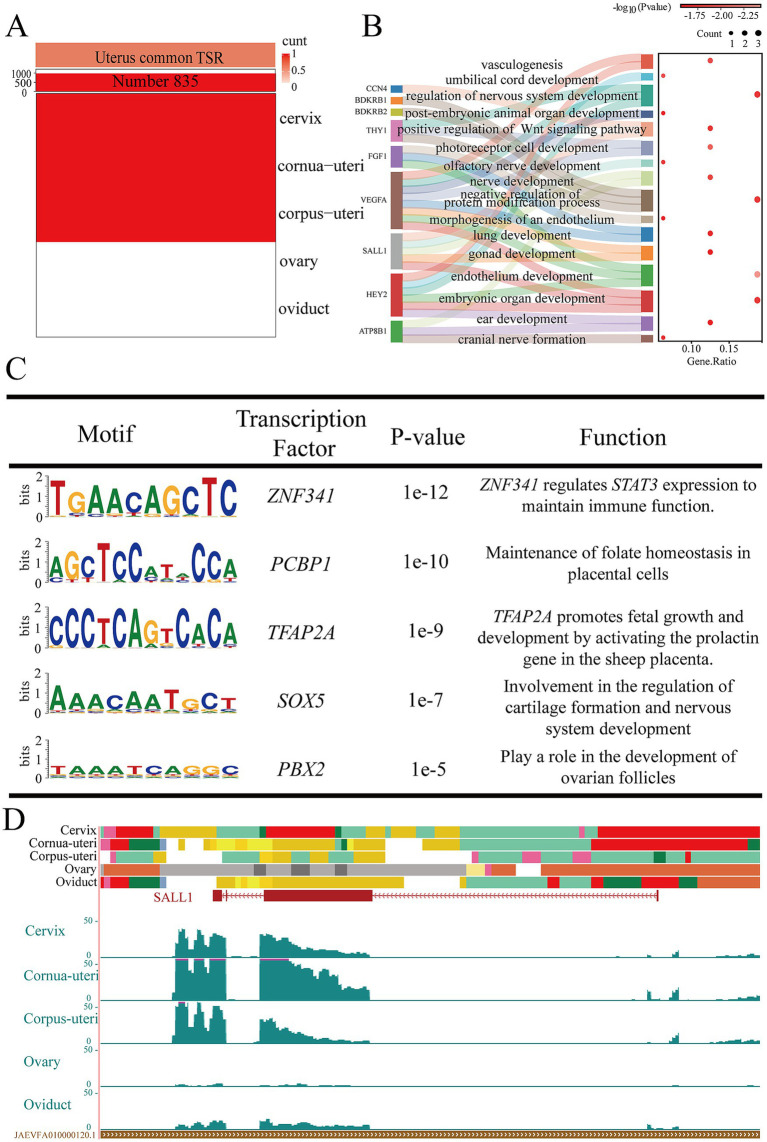
Uterine common tissue-specific strong enhancers (EnhA) and functional annotation. **(A)** The number and spatial distribution of EnhA in uterine common tissue, along with their enrichment patterns. **(B)** GO functional enrichment analysis based on the target genes of uterine common EnhAs. **(C)** Enrichment analysis of transcription factor motifs in uterine common tissue. **(D)** The chromatin state landscape and mRNA expression of the *SALL1* (chr14:19,060,302-19,081,400, Ramb_v2.0) locus across five tissues. The vertical scale of the UCSC track represents the normalized RNA-seq signal, ranging from 0 to 50.

In order to study the function of the uterus, we focused on EnhAs shared across the cervix, cornua uteri, and corpus uteri, identifying 835 shared uterine EnhAs (Figure 5A). GO analysis of the predicted target genes of these EnhAs revealed that the uterus contributes to the development of various organs during embryonic stages, including the lungs, ears, skin, nervous system, and blood vessels (Figure 5B). In the motif analysis, it was found that the uterus may contribute to the regulation of embryonic development through *TFAP2A* and *SOX5* (Figure 5C). To further explore the physiological relevance of uterine EnhAs, we used spalt like transcription factor 1 (*SALL1*) as a case study (Figure 5D).

### 3.6 Uterine common tissue-specific EnhA and functional annotation

**Figure 6:**
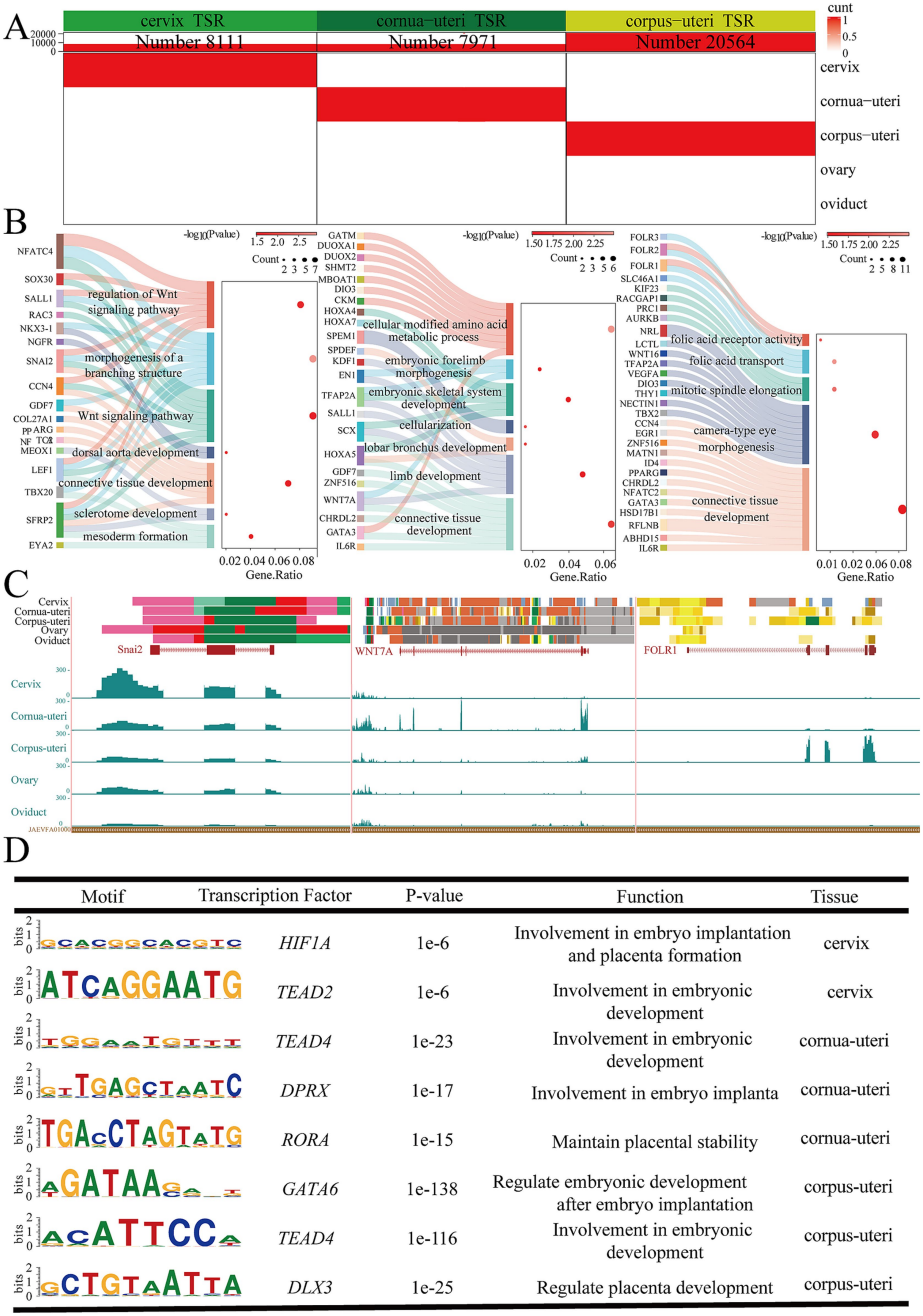
Uterus various tissue-specific strong enhancers (EnhA) and their functional annotation. **(A)** The number and spatial distribution of EnhA in cervix, cornua uteri, and corpus uteri tissue, along with their enrichment patterns. **(B)** GO functional enrichment analysis based on the target genes of cervix (left figure), cornua uteri (middle figure), and corpus uteri (right figure) EnhAs. **(C)** Enrichment analysis of transcription factor motifs in cervix (left figure), cornua uteri (middle figure), and corpus uteri (right figure) tissue. **(D)** The left figure shows the chromatin state landscape and mRNA expression of the *SNAI2* (chr9:32,949,607-32,955,054, Ramb_v2.0) locus across five tissues. The vertical scale of the UCSC track represents the normalized RNA-seq signal, ranging from 0 to 300. The middle figure shows the chromatin state landscape and mRNA expression of the *WNAT7A* (chr19:58,050,035-58,148,599, Ramb_v2.0) locus across five tissues. The vertical scale of the UCSC track represents the normalized RNA-seq signal, ranging from 0 to 10. The right figure shows the chromatin state landscape and mRNA expression of the *FOLR1* (chr15:50,521,446-50,538,520, Ramb_v2.0) locus across five tissues. The vertical scale of the UCSC track represents the normalized RNA-seq signal, ranging from 0 to 100.

The uterus is divided into three anatomical regions: cervix, cornua uteri, and corpus uteri. To explore the physiological functions of these distinct regions, separate analyses were performed for each, identifying 8,111 EnhAs in the cervix, 7,971 EnhAs in the cornua uteri, and 20,564 EnhAs in the corpus uteri (Figure 6A). The results of the GO analysis indicate that the functions associated with different regions vary. The cervix is primarily implicated in limb development, the cornua uteri mainly participates in mesoderm formation and the Wnt signaling pathway, and the corpus uteri maintains uterine function primarily through folic acid transport (Figure 6B). Motif enrichment analysis identified distinct transcription factors in each uterine region, including *HIF1A* and *TEAD2* in the cervix, *TEAD4*, *DPRX*, and *RORA* in the cornua uteri, and *GATA6*, *TEAD4*, and *DLX3* in the corpus uteri (Figure 6D). To further understand the physiological roles of these regions, we focused on several genes: snail family transcriptional repressor 2 (*SNAI2*) in the cervix, Wnt family member 7A (*WNT7A*) in the cornua uteri, and folate receptor alpha (*FOLR1*) in corpus uteri (Figure 6C).

### 3.7 Uterine common tissue-specific EnhA and functional annotation

**Figure 7:**
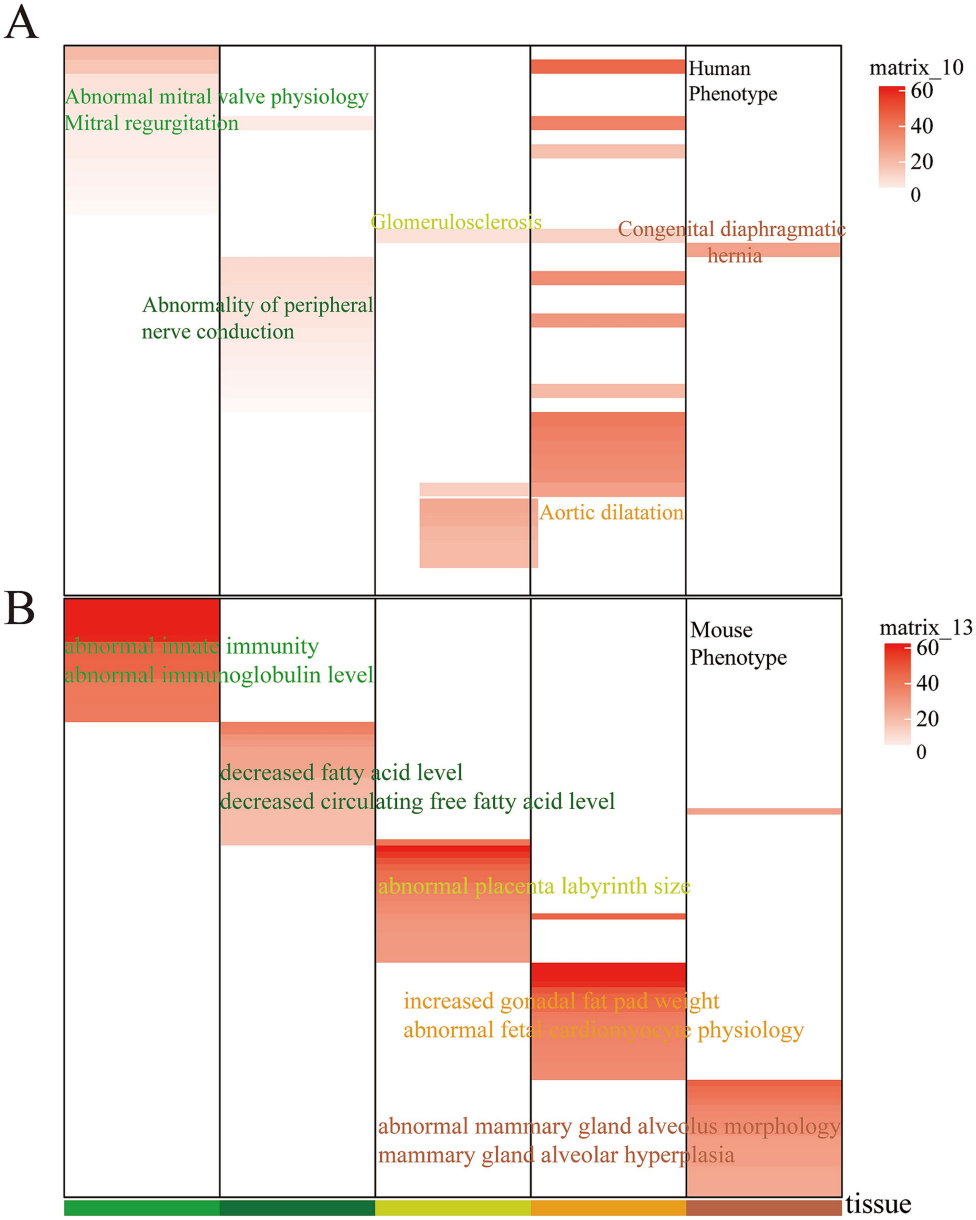
Human phenotype and mouse phenotype in tissue-specific strong enhancer. **(A)** Enrichment of specific human phenotypes. **(B)** Enrichment of specific mouse phenotypes. In the heatmap, the columns represent different tissues, the rows represent different phenotypes, and the color scale indicates the associated tissue for each entry.

Building on our previous work, which demonstrated that evolutionarily conserved enhancers retain tissue-specific biological functions and are linked to tissue-specific phenotypes in both humans and mice, we conducted an association analysis with human and mouse phenotypic data. In this study, our analysis revealed that cervix- and ovary-specific EnhAs are linked to mitral valve physiology in humans, while cornua uteri-specific EnhAs are associated with peripheral nerve conduction (Figure 7A). In the mouse phenotype context, corpus uteri-specific EnhAs are found to be associated with the placental labyrinth, and oviduct-specific EnhAs have been linked to mammary gland lobule morphology and alveolar development (Figure 7B).

## 4 Discussion

In this study, we constructed the first comprehensive map of regulatory elements in the reproductive tissues of sheep, covering five reproductive tissue types.^5^ A total of 1,680,172 regulatory elements, including 83,980 novel, tissue-specific EnhAs were identified and systematically characterized (26, 27). By analyzing the enriched pathways, associated transcription factors, and linked phenotypes (in humans and mice), we explored the role of these EnhAs in regulating reproductive functions across different tissues. The construction of this map marks a significant milestone for functional genomics annotation and offers valuable insights into the mechanisms underlying complex traits and diseases.

Tissue-specific functions are not predominantly located in conserved transcription start site regions (promoters) but are instead enriched in intergenic and intronic regions, often corresponding to enhancers (34). This study provides a pioneering investigation into the characteristics of tissue-specific EnhAs in sheep. In general, the regulation of tissue-specific functions is primarily achieved through two mechanisms. First, tissue-specific enhancers regulate putative target genes. GO analysis results indicate that the ovary-specific EnhAs target genes, such as *INHBA*, which play crucial roles in the process of ovulation. *INHBA* influences follicular development by modulating the synthesis of inhibin and activin, which impacts fertility in sheep (35, 36). *KITLG*, a gene deemed essential in the process of ovulation in mammals, is highly expressed in the oviduct (37, 38). The uterus, being the most significant reproductive organ, functions as a pivotal site for embryonic development. The *SALL1* gene, expressed in all three uterus regions, has been shown to play a role in the development of the gonads and embryonic organs (39, 40). Moreover, each uterine region exhibits distinct tissue-specific functions. *SNAI2*, a target gene of cervix-specific EnhAs, regulates morphogenetic events during embryonic development (41); *WNT7A*, a target gene of cornua uteri-specific EnhAs, plays a role in the development of the limbs and female reproductive system (42); and *FOLR1*, a target gene of corpus uteri-specific EnhAs, maintains fetal growth and vitality by regulating folate transport (43, 44). The second mechanism involves the recruitment of sequence-specific TFs, which are crucial for tissue development and the maintenance of tissue identity (45). In this study, a number of key TFs were found to be significantly enriched in tissue-specific EnhAs, including *OSR1* in the ovary (46), *FMR1* in the oviduct (47), *TEAD2* in the cervix (48, 49), *TEAD4* in the cornua uteri and corpus uteri (50–52), and *TFAP2A* (53), which was found to be common to all three uterus regions. These TFs have been shown to be crucial in the maintenance of normal embryonic development, through the regulation of ovarian physiology, trophoblast precursor differentiation, endometrial receptivity, and the expression of the placental prolactin gene. Consequently, the epigenomic map of sheep reproductive tissues provides insights into the complex gene regulation and functions of different tissues during reproduction and provides further insights.

Recent research in human epigenetics has highlighted the role of tissue-specific regulatory elements in complex traits and diseases (17, 54). Genetic variations associated with these traits are found to be particularly prevalent in open chromatin regions (55), and chromatin states (56). It has been demonstrated by preceding studies that the preservation of sequence across different species is a common method of identifying regulatory elements. Furthermore, it has been established that highly conserved regulatory elements are capable of regulating essential physiological processes (22). Cheng et al. discovered that regulatory elements are conserved across humans, mice, pigs, cattle, and sheep (24, 57). This conversation indicates the potential of domesticated animals as animal models for the study of human diseases. For instance, cattle can serve as models for human metabolic diseases (57), while pigs are valuable models for studying Alzheimer’s disease and inflammatory bowel disease (IBD) (22, 58). In this study, we established a correlation between abnormalities in the physiology of the mitral valve, fetal cardiomyocyte physiology, and placenta labyrinth morphology and the presence of EnhAs in sheep reproductive tissues. Based on these findings, we hypothesize that sheep may serve as an effective model for studying embryonic development and miscarriage. Furthermore, the epigenomic map of sheep reproductive tissues may serve as a valuable resource for advancing sheep as an animal model for human diseases.

In summary, a comprehensive and systematic approach was adopted to generate, collect, and analyze a substantial array of transcriptomic and epigenomic data from 60 datasets across five distinct reproductive tissues in sheep. Utilizing a multi-omics integrative approach, we constructed the first-ever epigenomic map of sheep reproductive tissue regulatory elements and investigated the functions of tissue-specific EnhAs. Furthermore, we integrated phenotype data from humans and mice to explore the potential application of sheep regulatory elements in diseases. Finally, a comprehensive catalogue of regulatory elements in sheep reproductive tissues was provided, with their potential roles in reproduction and disease highlighted.

## Data Availability

The raw data generated in this study has been deposited in the National Center for Biotechnology Information (NCBI, https://www.ncbi.nlm.nih.gov/) under the accession number PRJNA1237432. The RNA-seq and ATAC-seq processing pipelines are available on GitHub (https://github.com/kernco/functional-annotation), the CUT&Tag processing pipeline is available on GitHub (https://github.com/CebolaLab/CUTandTAG), and additional processing code is publicly accessible on GitHub (https://github.com/zhypan/Functional-Annotation-of-Pig and https://github.com/zhypan/FAANG_chicken).

## Footnotes

^1^ https://github.com/kernco/function-annotation

^2^ https://github.com/CebolaLab/CUTandTAG

^3^ https://github.com/theaidenlab/juicer

^4^ https://www.ncbi.nlm.nih.gov/datasets/genome/GCA_016772045.1/

^5^ https://genome.ucsc.edu/s/mengzhu/Sheep_Reproduction_GCF_016772045.1

## References

[1] Mazinani M Rude B. Population, world production and quality of sheep and goat products. Am J Anim Vet Sci. (2020) 15:291–9. doi: 10.3844/ajavsp.2020.291.299

[2] Li Y Liu Z Zhang W Zhu C Chen X Zhao Y. Effects of cloprostenol and oxytocin on ultrasonographic changes of uterine horns in postpartum primiparous Hu sheep. Anim Reprod Sci. (2025) 272:107651. doi: 10.1016/j.anireprosci.2024.107651, PMID: 39616723

[3] Gonçalves JD Dias JH Machado-Neves M Vergani GB Ahmadi B Pereira Batista RIT. Transcervical uterine flushing and embryo transfer in sheep: Morphophysiological basis for approaches currently used, major challenges, potential improvements, and new directions (alas, including some old ideas). Reprod Biol. (2024) 24:100920. doi: 10.1016/j.repbio.2024.100920, PMID: 38970979

[4] La Y Tang J Guo X Zhang L Gan S Zhang X. Proteomic analysis of sheep uterus reveals its role in prolificacy. J Proteome. (2020) 210:103526. doi: 10.1016/j.jprot.2019.103526, PMID: 31605788

[5] Fiorentino G Cimadomo D Innocenti F Soscia D Vaiarelli A Ubaldi FM. Biomechanical forces and signals operating in the ovary during folliculogenesis and their dysregulation: implications for fertility. Hum Reprod Update. (2023) 29:1–23. doi: 10.1093/humupd/dmac031, PMID: 35856663

[6] Juengel JL Reader KL Maclean PH Quirke LD Zellhuber-McMillan S Haack NA. The role of the oviduct environment in embryo survival. Reprod Fertil Dev. (2024) 36:RD23171. doi: 10.1071/RD23171, PMID: 38402905

[7] Clark EL Archibald AL Daetwyler HD Groenen MAM Harrison PW Houston RD. From FAANG to fork: application of highly annotated genomes to improve farmed animal production. Genome Biol. (2020) 21:285. doi: 10.1186/s13059-020-02197-8, PMID: 33234160 PMC7686664

[8] Moore JE Purcaro MJ Pratt HE Epstein CB Shoresh N Adrian J. Expanded encyclopaedias of DNA elements in the human and mouse genomes. Nature. (2020) 583:699–710. doi: 10.1038/s41586-020-2493-4, PMID: 32728249 PMC7410828

[9] Shlyueva D Stampfel G Stark A. Transcriptional enhancers: from properties to genome-wide predictions. Nat Rev Genet. (2014) 15:272–86. doi: 10.1038/nrg3682 24614317

[10] Carter B Zhao K. The epigenetic basis of cellular heterogeneity. Nat Rev Genet. (2021) 22:235–50. doi: 10.1038/s41576-020-00300-0, PMID: 33244170 PMC10880028

[11] Buniello A MacArthur JAL Cerezo M Harris LW Hayhurst J Malangone C. The NHGRI-EBI GWAS catalog of published genome-wide association studies, targeted arrays and summary statistics 2019. Nucleic Acids Res. (2019) 47:D1005–12. doi: 10.1093/nar/gky1120, PMID: 30445434 PMC6323933

[12] Tak YG Farnham PJ. Making sense of GWAS: using epigenomics and genome engineering to understand the functional relevance of SNPs in non-coding regions of the human genome. Epigenetics Chromatin. (2015) 8:57. doi: 10.1186/s13072-015-0050-4, PMID: 26719772 PMC4696349

[13] Giral H Landmesser U Kratzer A. Into the wild: GWAS exploration of non-coding RNAs. Front Cardiovasc Med. (2018) 5:181. doi: 10.3389/fcvm.2018.00181, PMID: 30619888 PMC6304420

[14] Carnielli CM Winck FV Paes Leme AF. Functional annotation and biological interpretation of proteomics data. Biochim Biophys Acta. (2015) 1854:46–54. doi: 10.1016/j.bbapap.2014.10.019, PMID: 25448015

[15] Cano-Gamez E Trynka G. From GWAS to function: using functional genomics to identify the mechanisms underlying complex diseases. Front Genet. (2020) 11:424. doi: 10.3389/fgene.2020.00424, PMID: 32477401 PMC7237642

[16] Márquez-Luna C Gazal S Loh P-R Kim SS Furlotte N Auton A. 23andMe research team, Price AL. Incorporating functional priors improves polygenic prediction accuracy in UK biobank and 23andMe data sets. Nat Commun. (2021) 12:6052. doi: 10.1038/s41467-021-25171-9, PMID: 34663819 PMC8523709

[17] Kundaje A Meuleman W Ernst J Bilenky M Yen A Kheradpour P. Integrative analysis of 111 reference human epigenomes. Nature. (2015) 518:317–30. doi: 10.1038/nature14248, PMID: 25693563 PMC4530010

[18] Zhang K Hocker JD Miller M Hou X Chiou J Poirion OB. A single-cell atlas of chromatin accessibility in the human genome. Cell. (2021) 184:5985–6001.e19. doi: 10.1016/j.cell.2021.10.024, PMID: 34774128 PMC8664161

[19] Gorkin DU Barozzi I Zhao Y Zhang Y Huang H Lee AY. An atlas of dynamic chromatin landscapes in mouse fetal development. Nature. (2020) 583:744–51. doi: 10.1038/s41586-020-2093-3 32728240 PMC7398618

[20] Yang H Luan Y Liu T Lee HJ Fang L Wang Y. A map of cis-regulatory elements and 3D genome structures in zebrafish. Nature. (2020) 588:337–43. doi: 10.1038/s41586-020-2962-9, PMID: 33239788 PMC8183574

[21] Son KH Aldonza MBD Nam A-R Lee K-H Lee J-W Shin K-J. Integrative mapping of the dog epigenome: reference annotation for comparative intertissue and cross-species studies. Sci Adv. (2023) 9:eade3399. doi: 10.1126/sciadv.ade3399, PMID: 37406108 PMC10321747

[22] Pan Z Yao Y Yin H Cai Z Wang Y Bai L. Pig genome functional annotation enhances the biological interpretation of complex traits and human disease. Nat Commun. (2021) 12:5848. doi: 10.1038/s41467-021-26153-7, PMID: 34615879 PMC8494738

[23] Pan Z Wang Y Wang M Wang Y Zhu X Gu S. An atlas of regulatory elements in chicken: a resource for chicken genetics and genomics. Sci Adv. (2023) 9:eade1204. doi: 10.1126/sciadv.ade1204, PMID: 37134160 PMC10156120

[24] Kern C Wang Y Xu X Pan Z Halstead M Chanthavixay G. Functional annotations of three domestic animal genomes provide vital resources for comparative and agricultural research. Nat Commun. (2021) 12:1821. doi: 10.1038/s41467-021-22100-8, PMID: 33758196 PMC7988148

[25] Peng S Dahlgren AR Donnelly CG Hales EN Petersen JL Bellone RR. Functional annotation of the animal genomes: An integrated annotation resource for the horse. PLoS Genet. (2023) 19:e1010468. doi: 10.1371/journal.pgen.1010468, PMID: 36862752 PMC10013926

[26] Davenport KM Massa AT Bhattarai S McKay SD Mousel MR Herndon MK. Characterizing genetic regulatory elements in ovine tissues. Front Genet. (2021) 12:628849. doi: 10.3389/fgene.2021.628849, PMID: 34093640 PMC8173140

[27] Zhang D Cheng J Li X Huang K Yuan L Zhao Y. Comprehensive multi-tissue epigenome atlas in sheep: a resource for complex traits, domestication, and breeding. iMeta. (2024) 3:e254. doi: 10.1002/imt2.254, PMID: 39742295 PMC11683475

[28] Liang G Lin JCY Wei V Yoo C Cheng JC Nguyen CT. Distinct localization of histone H3 acetylation and H3-K4 methylation to the transcription start sites in the human genome. Proc Natl Acad Sci. (2004) 101:7357–62. doi: 10.1073/pnas.0401866101, PMID: 15123803 PMC409923

[29] Creyghton MP Cheng AW Welstead GG Kooistra T Carey BW Steine EJ. Histone H3K27ac separates active from poised enhancers and predicts developmental state. Proc Natl Acad Sci. (2010) 107:21931–6. doi: 10.1073/pnas.1016071107, PMID: 21106759 PMC3003124

[30] Rada-Iglesias A. Is H3K4me1 at enhancers correlative or causative? Nat Genet. (2018) 50:4–5. doi: 10.1038/s41588-017-0018-3 29273804

[31] Kouzarides T. Chromatin modifications and their function. Cell. (2007) 128:693–705. doi: 10.1016/j.cell.2007.02.005, PMID: 17320507

[32] Fang L Liu S Liu M Kang X Lin S Li B. Functional annotation of the cattle genome through systematic discovery and characterization of chromatin states and butyrate-induced variations. BMC Biol. (2019) 17:68. doi: 10.1186/s12915-019-0687-8, PMID: 31419979 PMC6698049

[33] Yan Z Yang J Wei W-T Zhou M-L Mo D-X Wan X. A time-resolved multi-omics atlas of transcriptional regulation in response to high-altitude hypoxia across whole-body tissues. Nat Commun. (2024) 15:3970. doi: 10.1038/s41467-024-48261-w, PMID: 38730227 PMC11087590

[34] Foissac S Djebali S Munyard K Vialaneix N Rau A Muret K. Multi-species annotation of transcriptome and chromatin structure in domesticated animals. BMC Biol. (2019) 17:108. doi: 10.1186/s12915-019-0726-5, PMID: 31884969 PMC6936065

[35] Bao Y Yao X Li X Ei-Samahy MA Yang H Liang Y. INHBA transfection regulates proliferation, apoptosis and hormone synthesis in sheep granulosa cells. Theriogenology. (2021) 175:111–22. doi: 10.1016/j.theriogenology.2021.09.004 34537472

[36] Bao Y Li X El-Samahy MA Yang H Wang Z Yang F. Exploration the role of INHBA in Hu sheep granulosa cells using RNA-Seq. Theriogenology. (2023) 197:198–208. doi: 10.1016/j.theriogenology.2022.12.006, PMID: 36525859

[37] An XP Hou JX Gao TY Lei YN Song YX Wang JG. Association analysis between variants in *KITLG* gene and litter size in goats. Gene. (2015) 558:126–30. doi: 10.1016/j.gene.2014.12.058, PMID: 25550049

[38] Tang X Wang S Yi X Li Q Sun X. Identification of functional variants between Tong sheep and Hu sheep by whole-genome sequencing pools of individuals. Int J Mol Sci. (2024) 25:12919. doi: 10.3390/ijms252312919, PMID: 39684630 PMC11641353

[39] Nishinakamura R Takasato M. Essential roles of Sall1 in kidney development. Kidney Int. (2005) 68:1948–50. doi: 10.1111/j.1523-1755.2005.00626.x, PMID: 16221172

[40] de CJF Barrio R. Regulation and function of Spalt proteins during animal development. Int J Dev Biol. (2009) 53:1385–98. doi: 10.1387/ijdb.072408jd, PMID: 19247946

[41] Zhou W Gross KM Kuperwasser C. Molecular regulation of SNAI2 in development and disease. J Cell Sci. (2019) 132:jcs235127. doi: 10.1242/jcs.235127, PMID: 31792043 PMC12233911

[42] Lan L Wang W Huang Y Bu X Zhao C. Roles of Wnt7a in embryo development, tissue homeostasis, and human diseases. J Cell Biochem. (2019) 120:18588–98. doi: 10.1002/jcb.29217, PMID: 31271226

[43] Henderson GI Perez T Schenker S Mackins J Antony AC. Maternal-to-fetal transfer of 5-methyltetrahydrofolate by the perfused human placental cotyledon: evidence for a concentrative role by placental folate receptors in fetal folate delivery. J Lab Clin Med. (1995) 126:184–203. PMID: 7636392

[44] Nawaz FZ Kipreos ET. Emerging roles for folate receptor FOLR1 in signaling and cancer. Trends Endocrinol Metab. (2022) 33:159–74. doi: 10.1016/j.tem.2021.12.003, PMID: 35094917 PMC8923831

[45] Uhlén M Fagerberg L Hallström BM Lindskog C Oksvold P Mardinoglu A. Tissue-based map of the human proteome. Science. (2015) 347:1260419. doi: 10.1126/science.1260419, PMID: 25613900

[46] Lofrano-Porto A Pereira SA Dauber A Bloom JCB Fontes AN Asimow N. OSR1 disruption contributes to uterine factor infertility via impaired Müllerian duct development and endometrial receptivity. J Clin Invest. (2023) 133:e161701. doi: 10.1172/JCI161701, PMID: 37847567 PMC10688984

[47] Noto V Harrity C Walsh D Marron K. The impact of FMR1 gene mutations on human reproduction and development: a systematic review. J Assist Reprod Genet. (2016) 33:1135–47. doi: 10.1007/s10815-016-0765-6, PMID: 27432256 PMC5010819

[48] Godini R Fallahi H. Dynamics of transcription regulatory network during mice-derived retina organoid development. Gene. (2022) 813:146131. doi: 10.1016/j.gene.2021.146131, PMID: 34933077

[49] Guo Q Liu Q Wang N Wang J Sun A Qiao J. The function of nucleoporin 37 on mouse oocyte maturation and preimplantation embryo development. J Assist Reprod Genet. (2022) 39:107–16. doi: 10.1007/s10815-021-02330-x, PMID: 35022896 PMC8866631

[50] Sharma J Antenos M Madan P. A comparative analysis of hippo signaling pathway components during murine and bovine early mammalian embryogenesis. Genes. (2021) 12:281. doi: 10.3390/genes12020281, PMID: 33669396 PMC7920285

[51] Bačenková D Trebuňová M Čížková D Hudák R Dosedla E Findrik-Balogová A. In vitro model of human trophoblast in early placentation. Biomedicines. (2022) 10:904. doi: 10.3390/biomedicines10040904, PMID: 35453654 PMC9029210

[52] Liu B Yan J Li J Xia W. The role of BDNF, YBX1, CENPF, ZSCAN4, TEAD4, GLIS1 and USF1 in the activation of the embryonic genome in bovine embryos. Int J Mol Sci. (2023) 24:16019. doi: 10.3390/ijms242216019, PMID: 38003209 PMC10671747

[53] Limesand SW Anthony RV. Novel activator protein-2α splice-variants function as transactivators of the ovine placental lactogen gene. Eur J Biochem. (2001) 268:2390–401. doi: 10.1046/j.1432-1327.2001.02124.x 11298758

[54] Trynka G Sandor C Han B Xu H Stranger BE Liu XS. Chromatin marks identify critical cell types for fine mapping complex trait variants. Nat Genet. (2013) 45:124–30. doi: 10.1038/ng.2504, PMID: 23263488 PMC3826950

[55] Maurano MT Humbert R Rynes E Thurman RE Haugen E Wang H. Systematic localization of common disease-associated variation in regulatory DNA. Science. (2012) 337:1190–5. doi: 10.1126/science.1222794, PMID: 22955828 PMC3771521

[56] Ernst J Kheradpour P Mikkelsen TS Shoresh N Ward LD Epstein CB. Mapping and analysis of chromatin state dynamics in nine human cell types. Nature. (2011) 473:43–9. doi: 10.1038/nature09906, PMID: 21441907 PMC3088773

[57] Chen S Liu S Shi S Jiang Y Cao M Tang Y. Comparative epigenomics reveals the impact of ruminant-specific regulatory elements on complex traits. BMC Biol. (2022) 20:273. doi: 10.1186/s12915-022-01459-0, PMID: 36482458 PMC9730597

[58] Zhao Y Hou Y Xu Y Luan Y Zhou H Qi X. A compendium and comparative epigenomics analysis of cis-regulatory elements in the pig genome. Nat Commun. (2021) 12:2217. doi: 10.1038/s41467-021-22448-x, PMID: 33850120 PMC8044108

