# Supplementary material for: Regulatory element map of sheep reproductive tissues: functional annotation of tissue-specific strong active enhancers
Source: Front Vet Sci. 2025 Apr 16;12:1564148. doi: 10.3389/fvets.2025.1564148 (PMC12040938; doi:10.3389/fvets.2025.1564148)
Supplement: Supplementary file 1 [file Data_Sheet_1.docx]

Supplementary Material

# Supplementary Figures and Tables

## Supplementary Figure 1


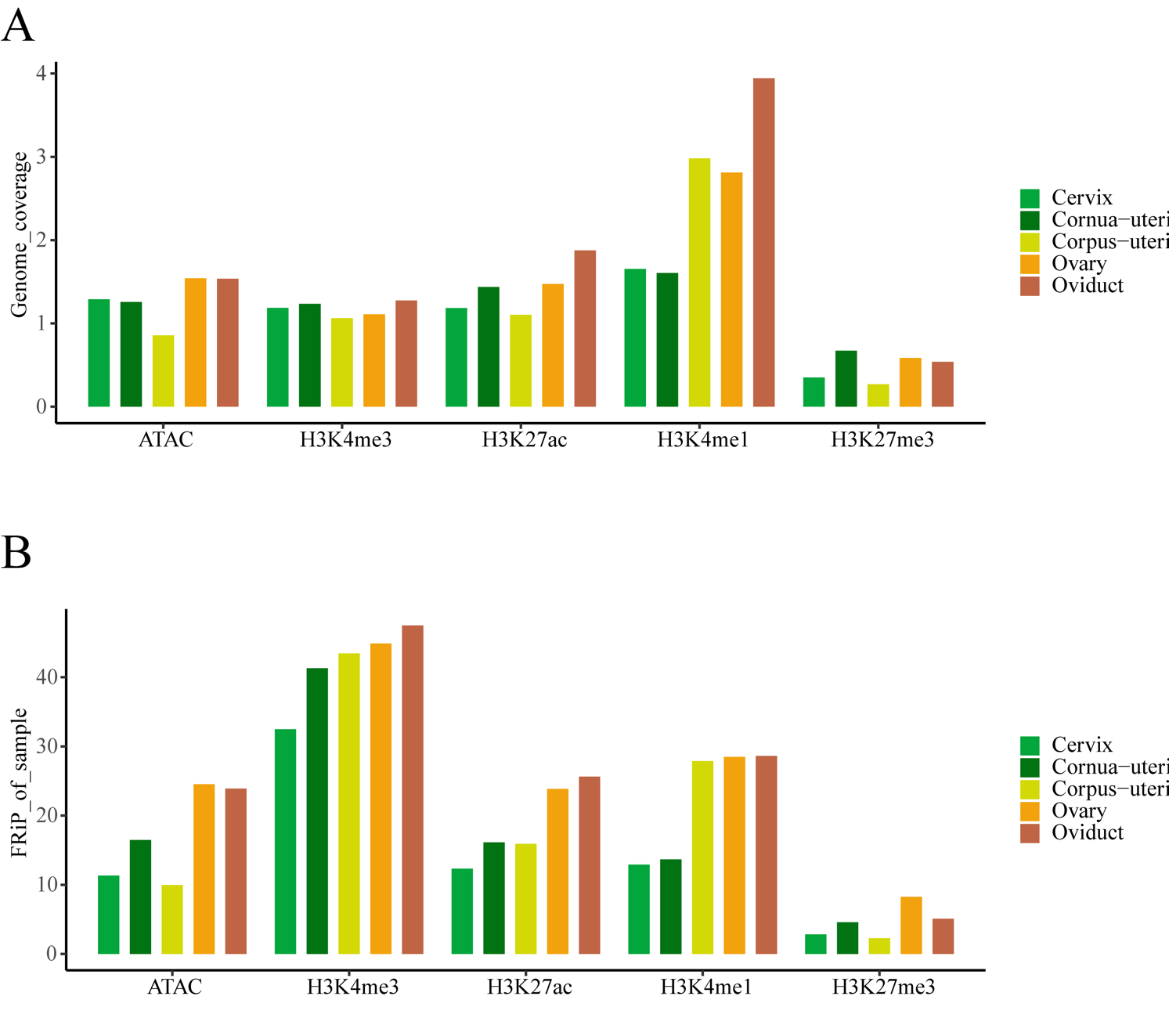


**Supplementary Figure 1.** (A) The average genomic coverage of peak regions for each epigenetic mark in each tissue. (B) The average Fraction of Reads in Peaks (FRiP) for epigenetic marks across five tissues.

## Supplementary Figure 2


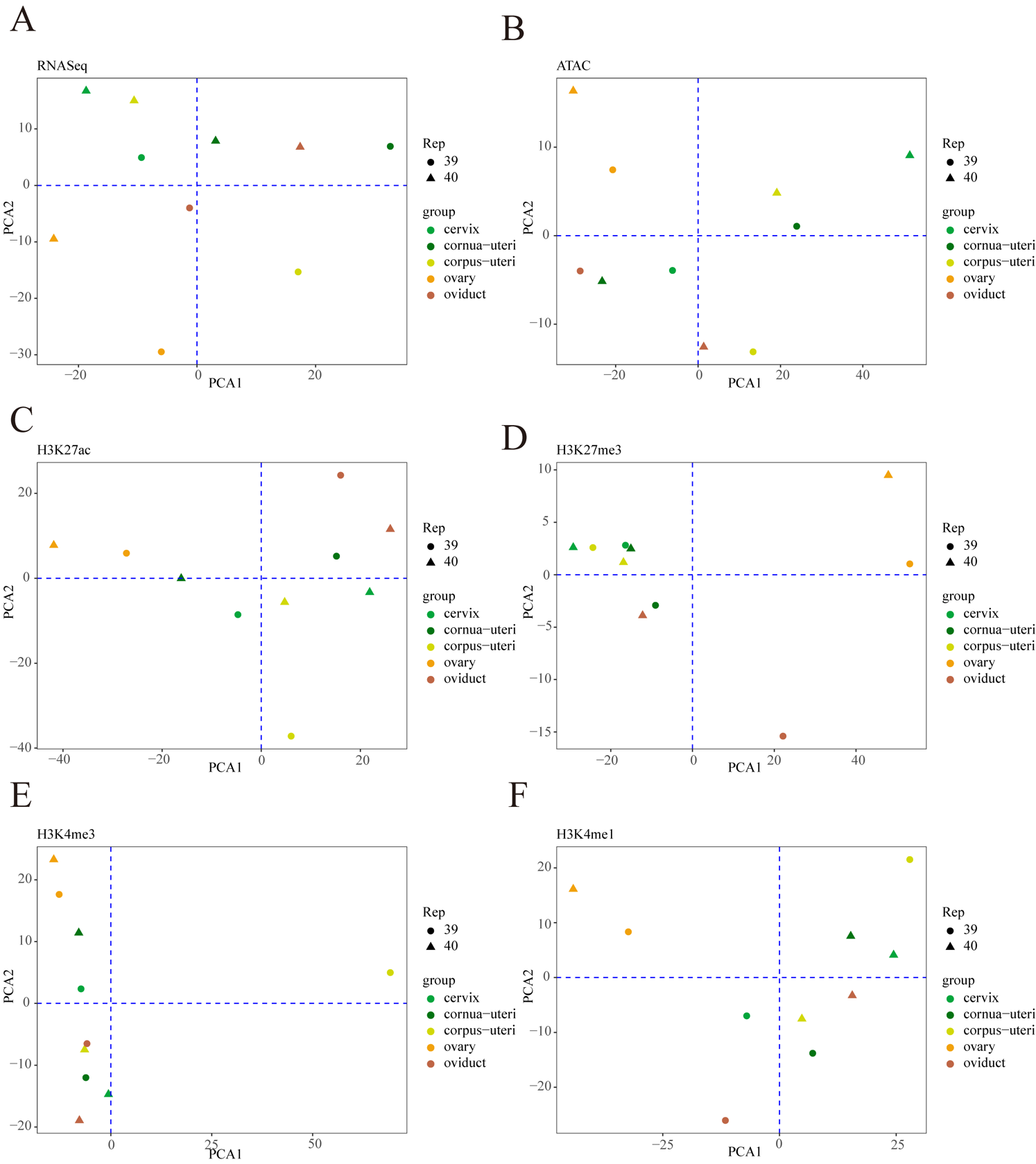


**Supplementary Figure 2.** (A-F) Principal component analysis (PCA) of epigenetic marks and RNA-seq data in five sheep reproductive tissues. The color and shape legends are consistent across all six panels.

## Supplementary Table 1

| Assay | Tissue | Replicate | Sequencing Depth | Mapped Fragment Number | Alignment Rate (%) | NRF | PBC1 | PBC2 | Peaks | FRIP | Genome Coverage (%) |
| --- | --- | --- | --- | --- | --- | --- | --- | --- | --- | --- | --- |
| ATAC | cervix | 39 | 37899711 | 36943003 | 98.83 | 0.61 | 0.63 | 2.78 | 59159 | 12.15 | 0.99 |
| H3K27ac | cervix | 39 | 20350557 | 19831759 | 97.45 | 0.75 | 0.75 | 3.90 | 44816 | 9.7 | 0.86 |
| H3K27me3 | cervix | 39 | 22199215 | 21643290 | 97.5 | 0.77 | 0.77 | 4.27 | 3686 | 1.12 | 0.06 |
| H3K4me1 | cervix | 39 | 21475938 | 20982319 | 97.7 | 0.74 | 0.74 | 3.75 | 72480 | 17.36 | 2.01 |
| H3K4me3 | cervix | 39 | 20589721 | 20047537 | 97.37 | 0.68 | 0.72 | 3.63 | 31305 | 31.75 | 1.08 |
| ATAC | cervix | 40 | 77410739 | 75483472 | 98.92 | 0.62 | 0.69 | 3.24 | 120742 | 10.51 | 1.58 |
| H3K27ac | cervix | 40 | 42347699 | 41178213 | 97.24 | 0.61 | 0.62 | 2.64 | 63304 | 14.96 | 1.51 |
| H3K27me3 | cervix | 40 | 31777986 | 31107943 | 97.89 | 0.70 | 0.71 | 3.38 | 41529 | 4.55 | 0.65 |
| H3K4me1 | cervix | 40 | 28023726 | 27409511 | 97.81 | 0.69 | 0.69 | 3.10 | 62018 | 8.46 | 1.29 |
| H3K4me3 | cervix | 40 | 51703228 | 50124421 | 96.95 | 0.54 | 0.60 | 2.59 | 35272 | 33.21 | 1.30 |
| ATAC | cornua-uteri | 39 | 27035878 | 25843929 | 97.03 | 0.80 | 0.82 | 5.76 | 43821 | 6.86 | 0.59 |
| H3K27ac | cornua-uteri | 39 | 25120905 | 24103350 | 95.95 | 0.72 | 0.73 | 3.70 | 55528 | 19.73 | 1.54 |
| H3K27me3 | cornua-uteri | 39 | 22802196 | 22203615 | 97.37 | 0.74 | 0.75 | 3.87 | 23497 | 3.62 | 0.46 |
| H3K4me1 | cornua-uteri | 39 | 20897932 | 20346671 | 97.36 | 0.73 | 0.73 | 3.61 | 74415 | 22.08 | 2.43 |
| H3K4me3 | cornua-uteri | 39 | 21561810 | 20967743 | 97.24 | 0.67 | 0.71 | 3.58 | 38549 | 39.11 | 1.23 |
| ATAC | cornua-uteri | 40 | 50198653 | 48816752 | 98.8 | 0.57 | 0.60 | 2.59 | 106584 | 26.08 | 1.92 |
| H3K27ac | cornua-uteri | 40 | 32865095 | 31966623 | 97.27 | 0.73 | 0.74 | 3.87 | 53301 | 12.53 | 1.33 |
| H3K27me3 | cornua-uteri | 40 | 45261220 | 44231874 | 97.73 | 0.66 | 0.68 | 3.05 | 45814 | 5.55 | 0.88 |
| H3K4me1 | cornua-uteri | 40 | 36346556 | 35530632 | 97.76 | 0.68 | 0.68 | 3.07 | 41526 | 5.28 | 0.78 |
| H3K4me3 | cornua-uteri | 40 | 34844402 | 33666484 | 96.62 | 0.58 | 0.66 | 3.18 | 29669 | 43.5 | 1.23 |
| ATAC | corpus-uteri | 39 | 36482332 | 35379100 | 98.44 | 0.76 | 0.78 | 4.48 | 69712 | 13.04 | 1.11 |
| H3K27ac | corpus-uteri | 39 | 20488069 | 19323853 | 94.32 | 0.72 | 0.74 | 3.82 | 43549 | 21.22 | 1.33 |
| H3K27me3 | corpus-uteri | 39 | 24089991 | 23421751 | 97.23 | 0.76 | 0.76 | 4.10 | 19492 | 2.61 | 0.39 |
| H3K4me1 | corpus-uteri | 39 | 20479915 | 19658854 | 95.99 | 0.70 | 0.72 | 3.59 | 69535 | 33.79 | 3.01 |
| H3K4me3 | corpus-uteri | 39 | 26686119 | 25861506 | 96.91 | 0.47 | 0.60 | 2.78 | 22230 | 55.36 | 0.90 |
| ATAC | corpus-uteri | 40 | 29600617 | 28861087 | 99.02 | 0.74 | 0.77 | 4.38 | 42520 | 6.9 | 0.60 |
| H3K27ac | corpus-uteri | 40 | 20708682 | 19305964 | 93.23 | 0.78 | 0.79 | 4.68 | 35520 | 10.58 | 0.88 |
| H3K27me3 | corpus-uteri | 40 | 22192367 | 21370989 | 96.3 | 0.78 | 0.80 | 4.95 | 8296 | 1.9 | 0.15 |
| H3K4me1 | corpus-uteri | 40 | 26073066 | 24631140 | 94.47 | 0.75 | 0.76 | 4.08 | 80942 | 21.97 | 2.95 |
| H3K4me3 | corpus-uteri | 40 | 20895667 | 19844883 | 94.97 | 0.72 | 0.76 | 4.46 | 31718 | 31.5 | 1.22 |
| ATAC | Ovary | 39 | 39280437 | 38208733 | 98.95 | 0.73 | 0.73 | 3.60 | 75243 | 21.75 | 1.53 |
| H3K27ac | Ovary | 39 | 22699748 | 22124405 | 97.47 | 0.69 | 0.70 | 3.40 | 56158 | 24.15 | 1.56 |
| H3K27me3 | Ovary | 39 | 19705447 | 19259279 | 97.74 | 0.69 | 0.70 | 3.38 | 22495 | 9.74 | 0.66 |
| H3K4me1 | Ovary | 39 | 20707514 | 20253844 | 97.81 | 0.70 | 0.71 | 3.42 | 90088 | 33.5 | 3.41 |
| H3K4me3 | Ovary | 39 | 27466422 | 26684806 | 97.15 | 0.59 | 0.67 | 3.31 | 29208 | 44.6 | 1.19 |
| ATAC | Ovary | 40 | 33063856 | 32136234 | 98.9 | 0.67 | 0.69 | 3.21 | 74679 | 27.33 | 1.55 |
| H3K27ac | Ovary | 40 | 20598646 | 20080664 | 97.49 | 0.68 | 0.70 | 3.44 | 46144 | 23.55 | 1.39 |
| H3K27me3 | Ovary | 40 | 19858600 | 19402276 | 97.7 | 0.73 | 0.74 | 3.75 | 17719 | 6.8 | 0.51 |
| H3K4me1 | Ovary | 40 | 20568589 | 20056885 | 97.51 | 0.68 | 0.69 | 3.15 | 56877 | 23.44 | 2.2106 |
| H3K4me3 | Ovary | 40 | 24232913 | 23501967 | 96.98 | 0.58 | 0.67 | 3.34 | 23034 | 45.13 | 1.02778 |
| ATAC | oviduct | 39 | 27712649 | 26826032 | 98.64 | 0.74 | 0.75 | 4.00 | 71362 | 22.17 | 1.26526 |
| H3K27ac | oviduct | 39 | 25034554 | 24287036 | 97.01 | 0.70 | 0.72 | 3.61 | 53734 | 21.6 | 1.58558 |
| H3K27me3 | oviduct | 39 | 19220941 | 18657801 | 97.07 | 0.77 | 0.78 | 4.46 | 14878 | 4.05 | 0.328426 |
| H3K4me1 | oviduct | 39 | 25212377 | 24536515 | 97.32 | 0.69 | 0.70 | 3.28 | 73706 | 26.81 | 2.80683 |
| H3K4me3 | oviduct | 39 | 30029518 | 28969069 | 96.47 | 0.57 | 0.67 | 3.39 | 24842 | 47.29 | 1.13545 |
| ATAC | oviduct | 40 | 41590693 | 40597036 | 98.94 | 0.59 | 0.61 | 2.65 | 103712 | 25.62 | 1.80581 |
| H3K27ac | oviduct | 40 | 30174097 | 29163341 | 96.65 | 0.69 | 0.71 | 3.64 | 66843 | 29.68 | 2.16468 |
| H3K27me3 | oviduct | 40 | 21147746 | 20665152 | 97.72 | 0.76 | 0.77 | 4.35 | 33713 | 6.15 | 0.747414 |
| H3K4me1 | oviduct | 40 | 39484478 | 38662880 | 97.92 | 0.65 | 0.65 | 2.87 | 142319 | 30.45 | 5.07891 |
| H3K4me3 | oviduct | 40 | 40369042 | 38945947 | 96.47 | 0.57 | 0.69 | 3.63 | 32000 | 47.66 | 1.41682 |

**Supplementary Table 1.** Note: Non-Redundant Fraction (NRF), PCR Bottlenecking Coefficient 1 (PBC1), PCR Bottlenecking Coefficient 2 (PBC2), Fraction of reads in peaks (FRiP) .
